# Development of Novel Patient-Derived Xenografts from Breast Cancer Brain Metastases

**DOI:** 10.3389/fonc.2017.00252

**Published:** 2017-11-02

**Authors:** María J. Contreras-Zárate, D. Ryan Ormond, Austin E. Gillen, Colton Hanna, Nicole L. Day, Natalie J. Serkova, Britta M. Jacobsen, Susan M. Edgerton, Ann D. Thor, Virginia F. Borges, Kevin O. Lillehei, Michael W. Graner, Peter Kabos, Diana M. Cittelly

**Affiliations:** ^1^Department of Pathology, University of Colorado Anschutz Medical Campus, Aurora, CO, United States; ^2^Department of Neurosurgery, University of Colorado Anschutz Medical Campus, Aurora, CO, United States; ^3^RNA Bioscience Initiative, University of Colorado Anschutz Medical Campus, Aurora, CO, United States; ^4^Department of Anesthesiology, University of Colorado Anschutz Medical Campus, Aurora, CO, United States; ^5^Department of Medicine, Division of Medical Oncology, University of Colorado Anschutz Medical Campus, Aurora, CO, United States

**Keywords:** patient-derived xenograft, brain metastases models, breast cancer, brain colonization, triple negative, HER2^+^

## Abstract

Brain metastases are an increasing burden among breast cancer patients, particularly for those with HER2^+^ and triple negative (TN) subtypes. Mechanistic insight into the pathophysiology of brain metastases and preclinical validation of therapies has relied almost exclusively on intracardiac injection of brain-homing cells derived from highly aggressive TN MDA-MB-231 and HER2^+^ BT474 breast cancer cell lines. Yet, these well characterized models are far from representing the tumor heterogeneity observed clinically and, due to their fast progression *in vivo*, their suitability to validate therapies for established brain metastasis remains limited. The goal of this study was to develop and characterize novel human brain metastasis breast cancer patient-derived xenografts (BM-PDXs) to study the biology of brain metastasis and to serve as tools for testing novel therapeutic approaches. We obtained freshly resected brain metastases from consenting donors with breast cancer. Tissue was immediately implanted in the mammary fat pad of female immunocompromised mice and expanded as BM-PDXs. Brain metastases from 3/4 (75%) TN, 1/1 (100%) estrogen receptor positive (ER^+^), and 5/9 (55.5%) HER2^+^ clinical subtypes were established as transplantable BM-PDXs. To facilitate tracking of metastatic dissemination using BM-PDXs, we labeled PDX-dissociated cells with EGFP-luciferase followed by reimplantation in mice, and generated a BM-derived cell line (F2-7). Immunohistologic analyses demonstrated that parental and labeled BM-PDXs retained expression of critical clinical markers such as ER, progesterone receptor, epidermal growth factor receptor, HER2, and the basal cell marker cytokeratin 5. Similarly, RNA sequencing analysis showed clustering of parental, labeled BM-PDXs and their corresponding cell line derivative. Intracardiac injection of dissociated cells from BM-E22-1, resulted in magnetic resonance imaging-detectable macrometastases in 4/8 (50%) and micrometastases (8/8) (100%) mice, suggesting that BM-PDXs remain capable of colonizing the brain at high frequencies. Brain metastases developed 8–12 weeks after ic injection, located to the brain parenchyma, grew around blood vessels, and elicited astroglia activation characteristic of breast cancer brain metastasis. These novel BM-PDXs represent heterogeneous and clinically relevant models to study mechanisms of brain metastatic colonization, with the added benefit of a slower progression rate that makes them suitable for preclinical testing of drugs in therapeutic settings.

## Introduction

Brain metastases are the most common form of brain cancer, exceeding the number of primary brain tumors by at least four times, and occurring in about 25% of all patients with cancer (1). Breast cancer is the second most common primary tumor responsible for brain metastasis (2, 3), especially from women with HER2^+^ and triple negative [TN, estrogen receptor negative (ER^−^), progesterone receptor negative (PR^−^), and HER2^−^] tumors (4–6). Brain metastases remain incurable and more than 80% of patients will die within a year of their brain-metastasis diagnosis (7, 8). Treating brain metastases has been particularly challenging due to unique anatomical and functional features in the brain. Therapies used to treat systemic metastases [e.g., trastuzumab for the treatment of breast tumors overexpressing HER2^+^, or chemotherapies used to treat triple negative breast cancers (TNBCs)] have limited ability to cross the blood–brain barrier (BBB) at effective doses, and often fail to decrease brain metastatic burden (8, 9). Thus, there is an urgent need for improved therapeutic approaches for breast cancer brain metastases.

A critical limitation to achieve better therapeutic strategies for brain metastasis has been the narrow set of experimental models to study brain metastasis pathophysiology. Development of symptomatic brain metastasis requires cancer cells to disseminate from the primary tumor, intravasate into blood vessels, survive in circulation, extravasate through the BBB, survive the neuroinflammatory response in the brain, and outgrow into large metastasis (10–12). Studying this complex process requires *in vivo* animal models that mimic early and late stages of brain metastatic colonization, produce brain metastases at high frequencies, and demonstrate moderate tumor progression necessary for the preclinical screening of drugs that could be used in preventive and therapeutic settings (13, 14). Until recently, brain metastasis studies relied primarily on intracardiac (ic) injection of brain-homing cells derived from murine 4T1 (4T1BR5), human TN MDA-MB-231 (231Br, 231/LM2-4) (15–17), and HER2^+^BT474 (BT474BR) cell lines (18). These models were developed by performing successive rounds of ic injection of breast cancer cell lines, which were then reisolated, cultured *in vitro* and then reinjected into nude mice (19). Although these brain metastatic cell lines are well characterized and produce brain metastases at high frequencies (20, 21), the rapid progression of metastatic burden in these models limits their usability for therapeutic testing of drugs. More importantly, these models do not fully represent the heterogeneity observed in breast tumors and their metastasis, which have emerged as critical factor in defining populations of patients that are likely to respond to a particular therapy.

During the past several years, researchers have developed transplantable models to grow primary breast tumors in the mammary fat pad of NOD/SCID/ILIIrg^−/−^ (NSG) mice, with the long-term goal of personalizing medicine (22–24). These PDXs retain intratumoral heterogeneity and have become a clinically relevant alternative to cell lines (23, 25, 26). Here, we report the development and characterization of eight novel human breast cancer patient-derived xenografts (BM-PDXs) from ER^+^, HER2^+^, and TN subtypes and a matching TN cell line, which retain tumor heterogeneity and brain metastatic potential. We demonstrate that ic injection of cells dissociated from BM-PDXs produce brain metastases at high frequencies, with metastases that elicit astroglia activation and growth around vessels in a similar fashion to breast cancer brain metastasis. These novel BM-PDXs represent heterogeneous and clinically relevant models to study mechanisms of brain metastatic colonization, with the added benefit of a slower progression rate that makes them suitable for preclinical testing of drugs in therapeutic settings.

## Materials and Methods

### Brain Metastases Transplantation and Establishment of Patient-Derived Xenografts

De-identified brain metastases and their clinical-pathological information (age, ER, PR, and HER2 status at the time of metastases resection, prior therapies, and survival) were obtained from consenting breast cancer patients undergoing neurosurgery. These samples were collected under approved IRB protocols at University of Colorado Anschutz Medical Campus. All animal studies were performed under approved University of Colorado Institutional Animal Care and Use Committee (IACUC) protocols.

Freshly removed brain metastasis samples were placed on sterile ice-cold DMEM and transported to the laboratory for transplantation into mice. Specimens that could not be immediately implanted were maintained at 4°C for no longer than 8 h. Female NSG mice, 6–8 weeks old were purchased from Jackson laboratories or bred at the UC Denver Center for Comparative Medicine breeding facility. Brain metastases were partitioned into 5–10 mm^3^ pieces, dipped into cultrex, and implanted in the fourth mammary fat pad of anesthesized mice using a 10-gage trochar. In one case, brain metastatic cells were collected from cerebrospinal fluid from a patient diagnosed with meningeal carcinomatosis. Here, cancer cells were collected by centrifugation and divided into two aliquots. One mouse was injected ic with cancer cells suspended in 100 μl PBS, another recipient was injected into the mammary fat pad with cancer cells resuspended in 50 μl cultrex. All mice were implanted with a silastic pellet providing slow release of 17β-estradiol (E2), as prior experience showed that E2 increases tumor uptake of breast cancer PDXs irrespective of tumor subtype. Tumors were palpated weekly to assess tumor take for up to 8 months postimplantation. Once palpable, tumor size was assessed weekly using caliper and volume estimated as length × width^2^/2. When tumors reached ~1.5 cm in any direction, mice were euthanized and tumors removed. Tumors were divided into several 10 mm^3^ pieces and reimplanted in the mammary fat pad of NSG mice, cryopreserved in 10% DMSO/90% FBS in liquid nitrogen, stored in trizol RNA extraction, and fixed in 10% formalin for paraffin embedding. BM-PDXs were considered established if they grew over two generations.

### Labeling of BM-PDXs

A subset of BM-PDXs were labeled with lentiviral particles expressing EGFP-luciferase as we described previously (27). Briefly, >1 cm^3^ tumors were resected from euthanized mice and digested in Accumax (Stemcell Tech) for 3 h at 30°C. Human cancer cells were separated from mouse stromal cells using a lineage cell depletion kit (MACS) and isolated breast cancer cells were plated in six-well ultralow attachment plates in DMEM-F12 media. Tumor cells were transduced with 30 MOI of lentiviral pHAGE-EF1aL-luciferase-UBC-GFP-W and GFP expression monitored for up to 48 h. Labeled tumor cells were then collected, washed, resuspended in 100 μl Cultrex basement membrane extract and injected in the mammary fat pad of NSG mice. Efficiency of transduction was assessed using luciferase activity imaging (IVIS) or GFP expression when tumors reached >1 cm^3^. Labeled BM-PDX were cryopreserved, fixed for immunohistological analysis, stored in trizol for RNA extraction, or transplanted into a new recipient.

### BM-F2-7 Cell Line Derivation and Culture

A cell line (F2-7) was derived from triple-negative BM-PDX. For this, tumor cells were dissociated from BM-PDX F2-7 using Accumax, and dissociated cells were plated in ultralow attachment six-well plates in DMEM-F12 supplemented with 10% of FBS, 1 μg/ml hydrocortisone, 100 ng/ml of cholera toxin, and 1 nM of insulin. After 4 weeks of growth in suspension, human cells free of fibroblasts were plated in collagen-I coated dishes, and purity validated by immunohistochemistry. F2-7 cells were labeled with GFP-luciferase as described for BM-PDXs. Short-Tandem Repeat analysis was performed in the established cell line and deposited at University of Colorado Tissue Culture Core facility for validation and future reference.

### RNA Sequencing of BM-PDX and F2-7 Cell Line

High-throughput RNA sequencing from a cell line derivative (F2-7) and a selected set of BM-PDXs before and after labeling with EGFP-luciferase was performed. RNA was isolated from tumor samples using trizol followed by RNA cleanup using RNEeasy MinElute Cleanup kit (Qiagen), and RNA concentration was measured in a Nanodrop 2000 (Thermo Scientific). The Genomics and Microarray core facility at the University of Colorado AMC performed RNA quality control using an Agilent 2100 Bioanalyzer, and prepared RNA-seq libraries using the Illumina TruSeq Stranded mRNA LT Sample Prep Kit. The resulting libraries were sequenced on an Illumina HiSeq 2500 system (1 × 125 bp). After demultiplexing, the resulting reads were trimmed with cutadapt to remove 3′ adaptor sequences and low quality 3′ bases (Q < 10). The trimmed reads were then aligned to both the human (hg19/GRCh37) and mouse (mm10) genomes using Tophat2 (28). Reads were then assigned to either the human or mouse genome using disambiguate (29) and ambiguous reads were discarded. Unambiguous reads were assigned to features using Rsubread (30) and normalized read counts were produced using the rlog function in DESeq2 (31). The GEO Accession number for this data is GSE104020.

### Experimental Brain Metastasis Using BM-PDXs

Two tumors from E22-1 BM-PDXs grown in the mammary fat pad were excised at necropsy, cut into 2 mm^2^ pieces and dissociated using Accumax for 3 h. The digestion was stopped using DMEM/F12 10% FBS, and single cells isolated by filtering through 100 and 70 μm mesh filters. Viable cells were counted using trypan blue exclusion and 250,000 cells resuspended in 100 μl PBS were injected in the left cardiac ventricle of recipient female NSG mice (*n* = 8). Brain metastatic burden was assessed using T1/T2 contrast magnetic resonance imaging (MRI) 8 weeks after ic injection, and mice were euthanized under CO_2_ asphyxiation at 8 or 12 weeks after ic injection as mice developed signs of CNS metastatic burden. In all cases, brains were removed at necropsy and brain hemispheres embedded in OCT and stored at −80°C until sectioning. Micrometastases were visualized with H&E and/or pan cytokeratin (PanCK) staining in six serial sections (10 μm thick), one every 300 μm in a sagittal plane through the right hemisphere of the brain.

### Magnetic Resonance Imaging

To non-invasively detect and quantify brain metastatic colonization, brain MR scans were acquired using a Bruker 4.7 T PharmaScan and a bird-cage radio frequency 36 mm coil (Bruker Medical, MA, USA). Animals were injected *via* tail vain with 0.4 mmol/kg gadolinium contrast Multihance (gadobenate dimeglumine, Bracco Diagnostic) and anesthetized with 2–2.5% isoflurane. High-resolution rapid acquisition with relaxation enhancement (RARE) T2-weighted images with fat suppression were obtained (TR/TE = 4,000/80 ms) followed by a multislice multiecho (MSME) T1-weighted sequence (TR/TE = 700/11 ms). All images were obtained in the axial plane, with the field of view of 3 cm, slice thickness 1 mm, number of slices 16, matrix size 256 × 256. In-plane resolution was 90 μm. T1-weighted MSME images were acquired as well to confirm metastatic lozation. All images were acquired and analyzed (T2-MRI for lesion numbers and diameters) using Bruker ParaVision (v4.3) software.

### Immunohistochemistry in PDXs and Experimental Brain Metastasis

Tumors were removed from animals and fixed in 10% buffered formalin. Tissue was processed, paraffin embedded, and cut into 5-μm sections. After high-temperature antigen retrieval in citrate buffer, sections were stained with rabbit anti-epidermal growth factor receptor (anti-EGFR, Cell Signaling), rabbit monoclonal anti-C-erB-2 (SP3, Neomarkers), mouse monoclonal antibody anti-cytokeratin 5 (anti-CK5, Vector), rabbit polyclonal anti-PR (DAKO, Carpinteria, CA, USA), and rabbit polyclonal anti-ERα (SP1, Thermofisher). Sections were counterstained with hematoxylin and mounted. Representative photographs were taken under a light microscope at ×20 magnification.

Dual immunofluorescence of brain metastasis was performed 10-μm sections from frozen unfixed-OCT embedded brains. Sections were fixed in acetone and stained with a mouse monoclonal antibody specific for human cytokeratins (Pan-CK, MNF116, Dakocytomation, Glostrup Denmark); in combination with rat anti-GFAP (Invitrogen, CA, USA); rabbit anti-collagen IV (Millipore). Secondary antibodies were anti-mouse Alexafluor-488 or anti-rabbit Alexafluor-565 or anti-Rat-Alexafluor-594 (all from Invitrogen/Thermofisher, CA, USA). Nuclei were stained with 1 μg/ml 4′,6-diamidino-2-phenylindole (DAPI) in methanol for 10 min at room temperature. Photographs were taken under ×20 magnification for the same field using the UV, FITC, and TRITC filters.

## Results

### Establishment of BM-PDXs in Relation to Breast Cancer Subtypes

**Figure 1:**
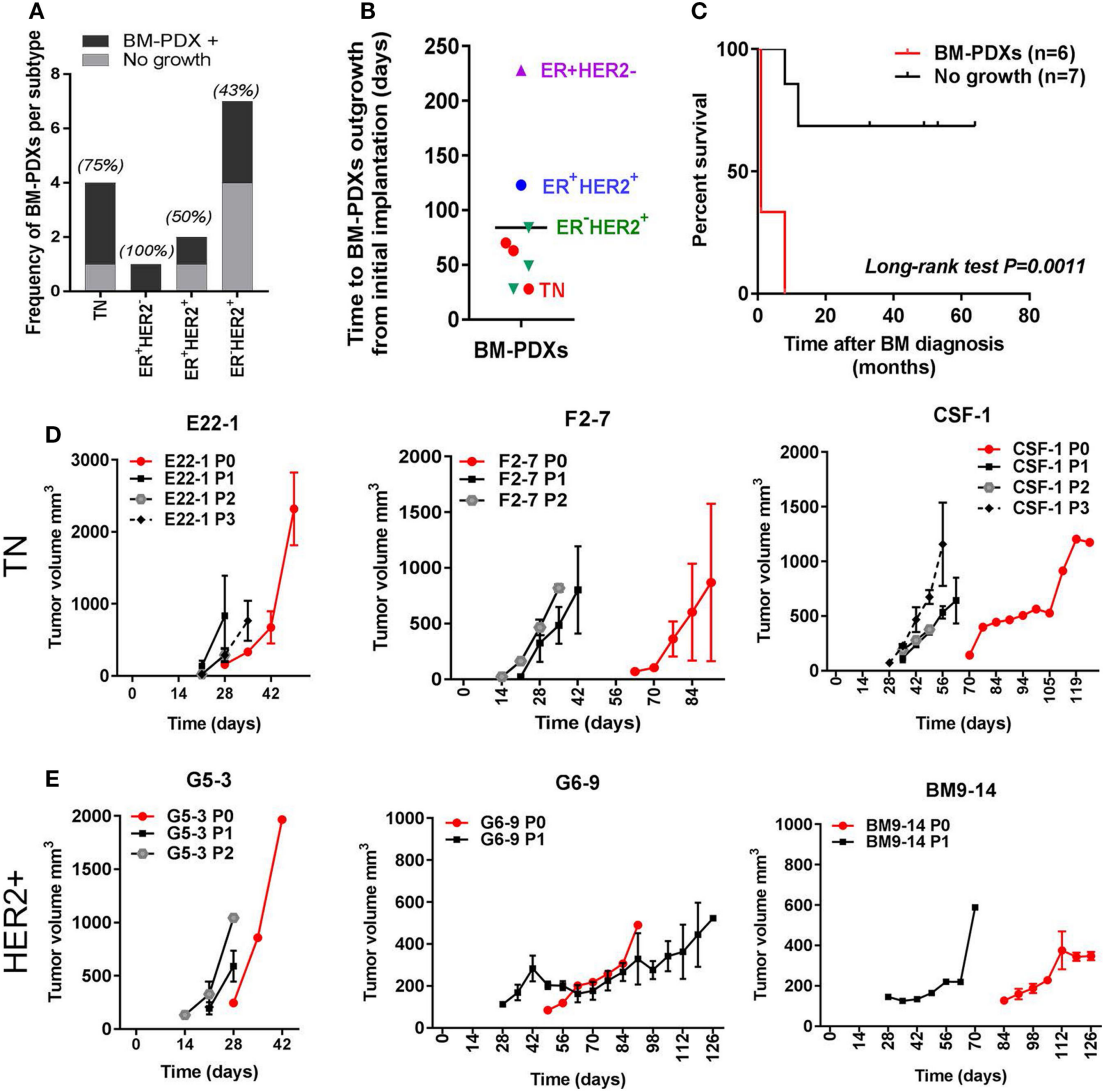
Establishment of brain-metastases-patient-derived xenografts (BM-PDXs) from breast cancer. PDXs were established in NOD/SCID/ILIIrg^−/−^ mice, and subsequently propagated *via* direct transplantation of solid tumor pieces into new recipient mice. Tumors were grown under continuous estrogen supplementation. **(A)** BM-PDXs established as a function of breast cancer subtypes. Graph depicts the number (bars) and percentage of BM-PDXs established (BM-PDXs^+^) per tumor subtype compared to tumors that did not grow after 8 months of implantation. **(B)** Time in days from initial implantation to outgrowth as measurable tumors (~62.5 mm^3^) for all PDXs, colors indicate breast cancer subtypes. **(C)** Survival (months) after brain metastases diagnosis of patients with breast cancer whose surgical samples had *in vivo* tumorigenic potential (*n* = 6), or not (*n* = 7). Log-rank (Mantel-Cox) test *P* value is shown. **(D,E)** Tumor growth after implantation (P0, red lines), and subsequent passaging (P1, P2, P3, black/gray lines) in **(D)** TN and **(E)** ER^−^HER2^+^ BM-PDXs. For P0, 1–2 tumors were implanted in a single recipient. For P1-P3, data shows average tumor volume from two tumors in 1–2 recipient mice.

We implanted a total of 14 brain metastases specimens from TN (*n* = 4), ER^+^HER2^−^ (*n* = 1), ER^+^HER2^+^ (*n* = 2), and ER^−^HER2^+^ (*n* = 7) breast cancer subtypes. From these, 8 (57.2%) successfully established as BM-PDXs, defined as those tumors that grew as xenografts in NSG mice at least in one consecutive passage and maintained expression of clinical markers from original patient sample. The frequency of BM-PDXs uptake varied among subtypes with a non-significant trend toward highest take rate in TNBC (3/4 BM-PDXs, 75%) and lower take rate in ER^−^HER2^+^ (3/7, 43%) (Figure 1A). The overall clinical–pathological characteristics of the brain metastases successfully established as BM-PDXs are presented in Table 1. Among HER2^+^BM-PDXs, two specimens had prior history of TNBC but their brain metastasis were diagnosed as HER2^+^; these were classified as HER2^+^BM-PDXs. Time to xenograft tumor formation for each BM-PDX at initial implantation ranked between 28 and 223 days, with an average of 84 days (Figure 1B). Similar to prior reports, *in vivo* tumorigenic potential of explanted brain metastases (tumors that grew as BM-PDXs) was correlated with decreased survival of their donor patients (*P* = 0.0011) (Figure 1C). Tumor progression in initial PDX (P0) and subsequent *in vivo* passaging (P1 to P3) for a TN and HER2^+^BM-PDXs is shown in Figures 1D,E. In one case, an ER-HER2^+^ brain metastases was implanted but an inguinal tumor developed suddenly after 70 days, away from the implantation site. This tumor lacked HER2, EGFR, or CK5 and upon transplantation grew into a large mass within 2 weeks (data not shown). As this suggested either loss of human epithelial markers or most likely, outgrowth of a murine tumor, we did not consider this a successful PDX and excluded it from further analysis.

**Table 1:** Clinical–pathological characteristics of BM-PDXs.

Clinical features of BM-PDXs						
			At brain metastasis diagnosis			
PDX	Primary tumor Dx	BM-Dx	Age	ER	PR	HER2
E22-1	TN	TN	58	0	0	0
F2-7	TN	TN	38	0	0	0
BM14-9	ER^−^HER2^+^	ER^−^HER2^+^	43	0	0	95% (3+)
G5-3	TN^a^	ER^−^HER2^+^	63	0	5% (1+)	30% (2+), FISH^+^
G6-9	TN^a^	ER^−^HER2^+^	53	0	0	40% (2+)
G7-1	ER^+^HER2^+^	ER^+^HER2^+^	59	65% (2+)	70% (2+)	45% (2+), FISH^+^
G13-1	ER^+^HER2^−^	ER^+^HER2^−^	63	70% (3+)	0	0
CSF-1^b^	TN	TN	ND	ND	ND	ND

*Numbers represent % of positive cells followed by intensity score (in brackets). HER2^+^ is defined as >10% and ≥(2+). ER^+^ is defined as > 1%*.

*^a^Primary tumors with history of TNBC that had converted to HER2^+^ at brain metastasis*.

*^b^Sample obtained from cerebrospinal fluid, no pathology report at metastatic site*.

*All patients had been treated with taxanes by the time of brain metastases*.

### Preservation of Clinical Markers in BM-PDXs Over Multiple Passaging, Cell Dissociation, and Viral-Mediated Transduction

**Figure 2:**
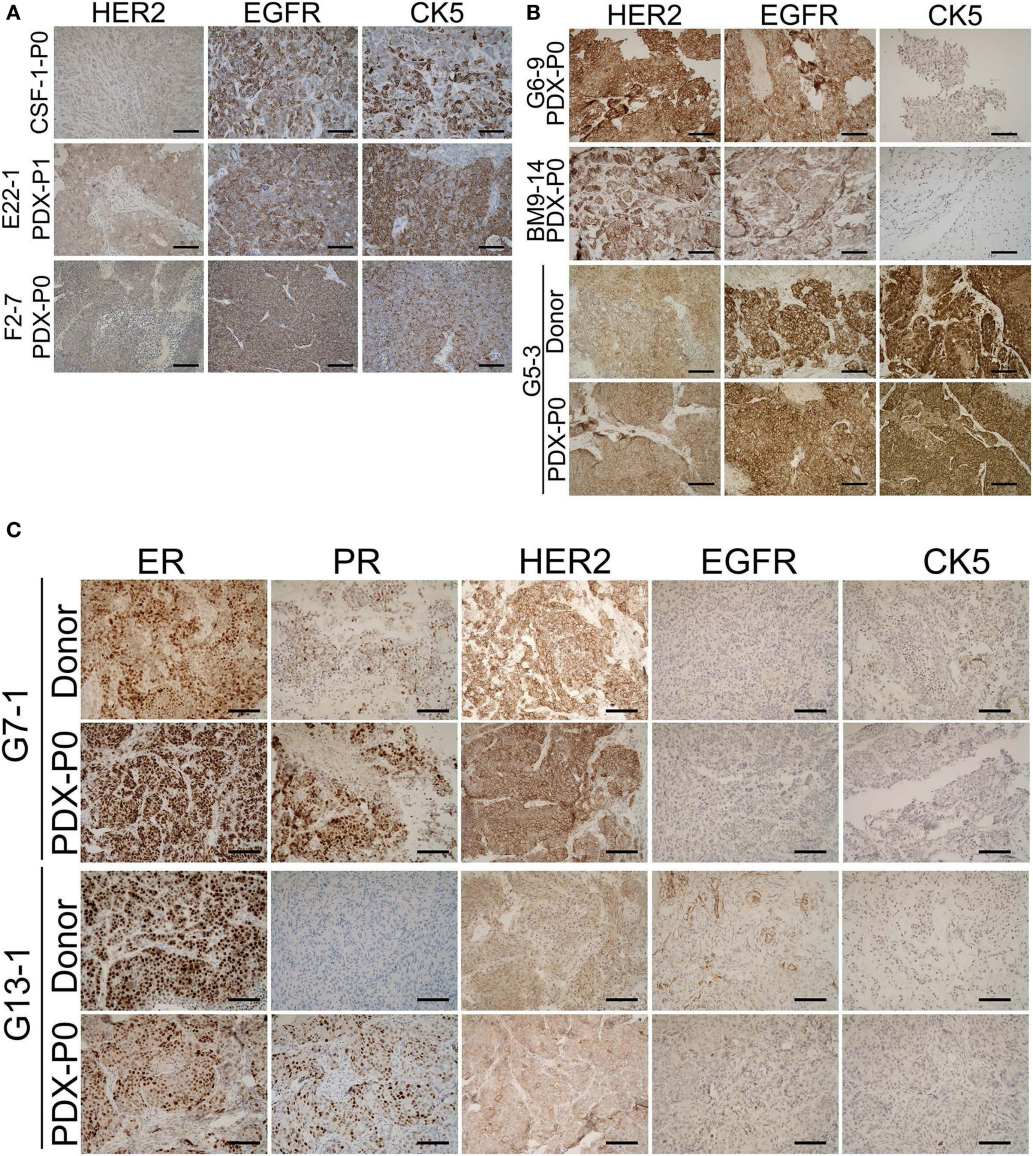
Retention of estrogen receptor (ER), progesterone receptor (PR), HER2, epidermal growth factor receptor (EGFR), and cytokeratin (CK) expression in brain-metastases-patient-derived xenografts (BM-PDXs). Sections of BM-PDXs were stained by IHC for ER, PR, HER2, EGFR, and CK5 at first passage (P0) and compared to donor tumor (when available). **(A)** Expression of EGFR, HER2, and CK5 in triple negative (TN) BM-PDXs (ER, PR, negative, not shown). **(B)** Expression of HER2, EGFR, and CK5 in HER2^+^BM-PDXs in donor and BM-PDXs P0. **(C)** Expression of ER, PR, HER2, EGFR, and CK5 in ER^+^HER2^+^BM-PDXs and their donor counterparts. Scale bars, 100 μm.

To determine whether outgrowth of BM-PDXs in the mammary fat pad retained key clinical features of brain metastases donors, we stained sections of BM-PDXs (P0-P1) for ER, PR, and HER2, and—when available—we compared them to clinical specimens at the time of implantation. TNBC brain metastases lack these markers but frequently express EGFR and CK5 (32). Thus, we added these to our validation panel. As show in Figure 2A, TN BM-PDXs expressed EGFR and CK5. For these samples there was no matching donor sample to compare, but lacked ER, PR, and HER2 as expected from TN tumors. ER-HER2^+^BM-PDXs retained HER2, EGFR, and CK5 (Figure 2B), and ER^+^BM-PDXs retained ER, HER2, EGFR, and CK5 expression similar to the donor sample (Figure 2C). Surprisingly the two ER^+^BM-PDXs (G7-1, G13-1), showed increased PR expression as compared to the donor samples (Figure 2C), suggesting that ER is functional in these BM-PDXs and that E2-supplementation in mice upregulates PR in ER^+^BM-PDXs.

**Figure 3:**
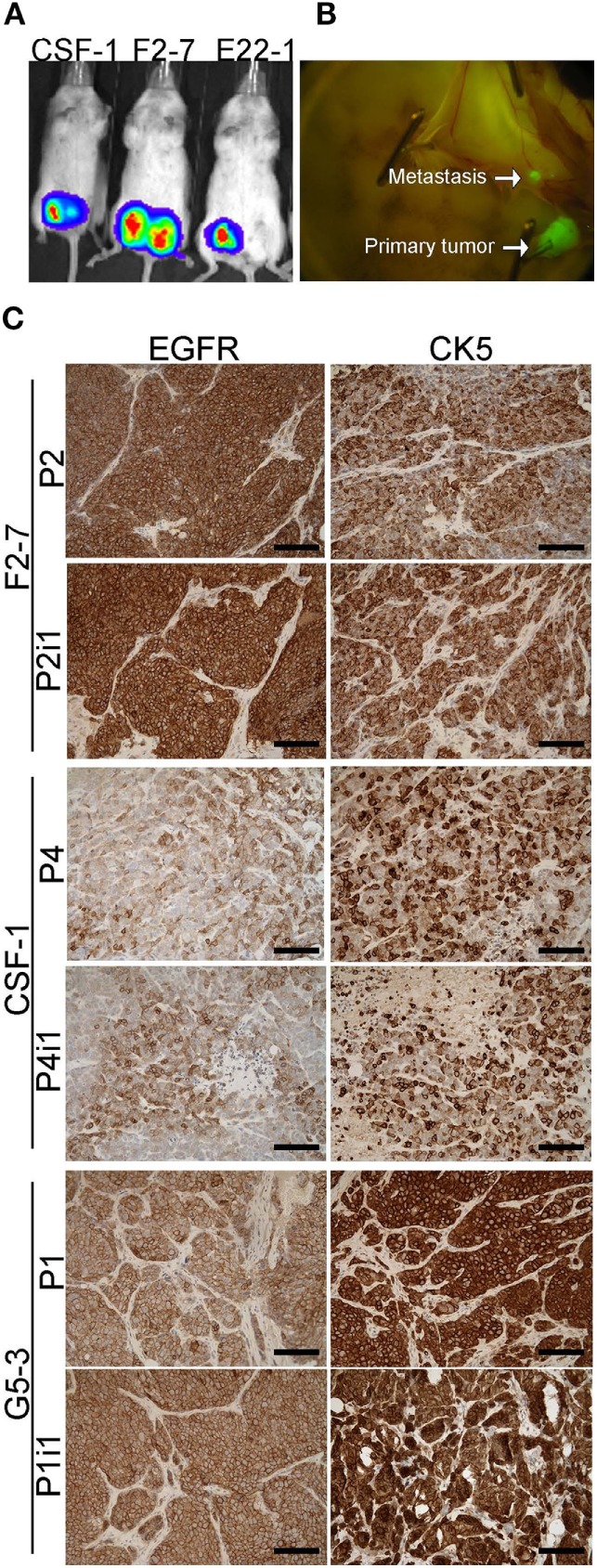
GFP-Luciferase-labeled brain-metastases-patient-derived xenografts (BM-PDXs) retain expression of epidermal growth factor receptor (EGFR), cytokeratin 5 (CK5), and HER2. **(A)** Luciferase imaging (IVIS) of NSG mice carrying GFP-luciferase BM-PDXs F2-7, CSF-1, and G5-3 in the mammary fat pad. **(B)** Spontaneous metastases to lymph node in G5-3 GFP-luc carrying mice. **(C)** Expression of EGFR and CK5 in sections of F2-7, CSF-1, and G5-3 BM-PDXs before (P1, P2) and after GFP-luciferase labeling (P2-I1, P1-I1). Scale bars, 100 μm.

To assess whether GFP and luciferase labeling would allow *in vivo* imaging of these BM-PDXs, we dissociated cells from >1 cm^3^ xenografts BM-PDXs F2-7, CSF-1, and G5-3 and transduced them with high titer viral particles of a GFP-luciferase vector as described (27). Labeled cells were regrown in the mammary fat pad of NSG mice and tumor labeling was assessed by measuring luciferase activity (IVIS) and GFP expression (Figure 3A). Spontaneous metastases to surrounding areas were detected in some mice during tumor excision (Figure 3B), but without spontaneous metastases to brain or other organs. Since tumor cell dissociation and cell transduction with lentiviral vector might results in selection of subclones of the original tumor, we assessed whether labeled PDXs recapitulated the heterogeneity observed in parental PDXs. IHC staining showed that labeled BM-PDXs retained expression of EGFR, CK5, and HER2 (Figure 3C), suggesting that dissociated/labeled cells are capable of reconstituting tumor heterogeneity of BM-PDXs.

**Figure 4:**
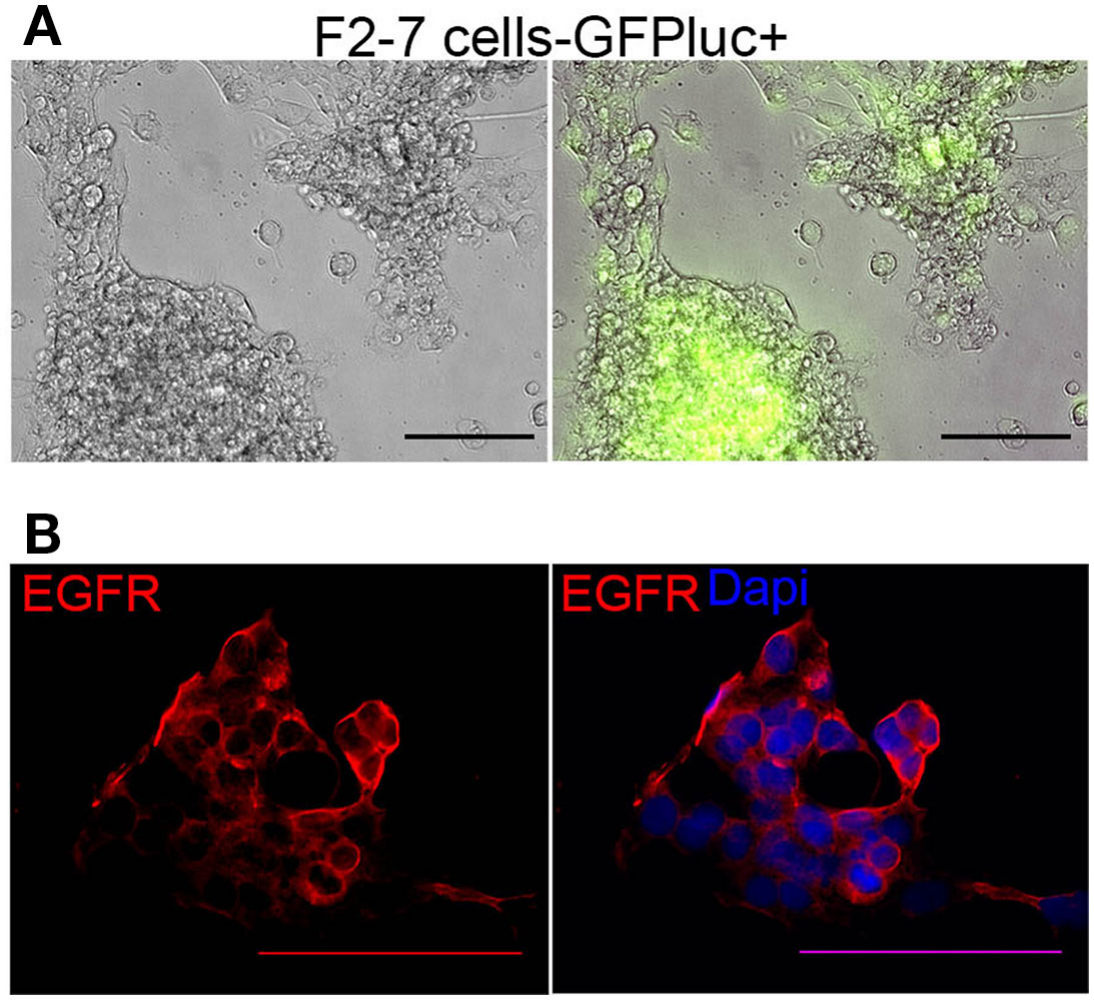
Characteristics of F2-7 BM-cell line *in vitro*. **(A)** F2-7 cells were labeled *in vitro* with GFP-luciferase and cultured in collagen-I coated plates. *Left*: Brightfield, *right*: GFP expression of live cells. **(B)** Immunofluorescence staining shows epidermal growth factor receptor (EGFR) (red) expression in F2-7 cells grown in coverslips. 4′,6-diamidino-2-phenylindole (DAPI) labels nuclei. Scale bars are 100 μm.

**Figure 5:**
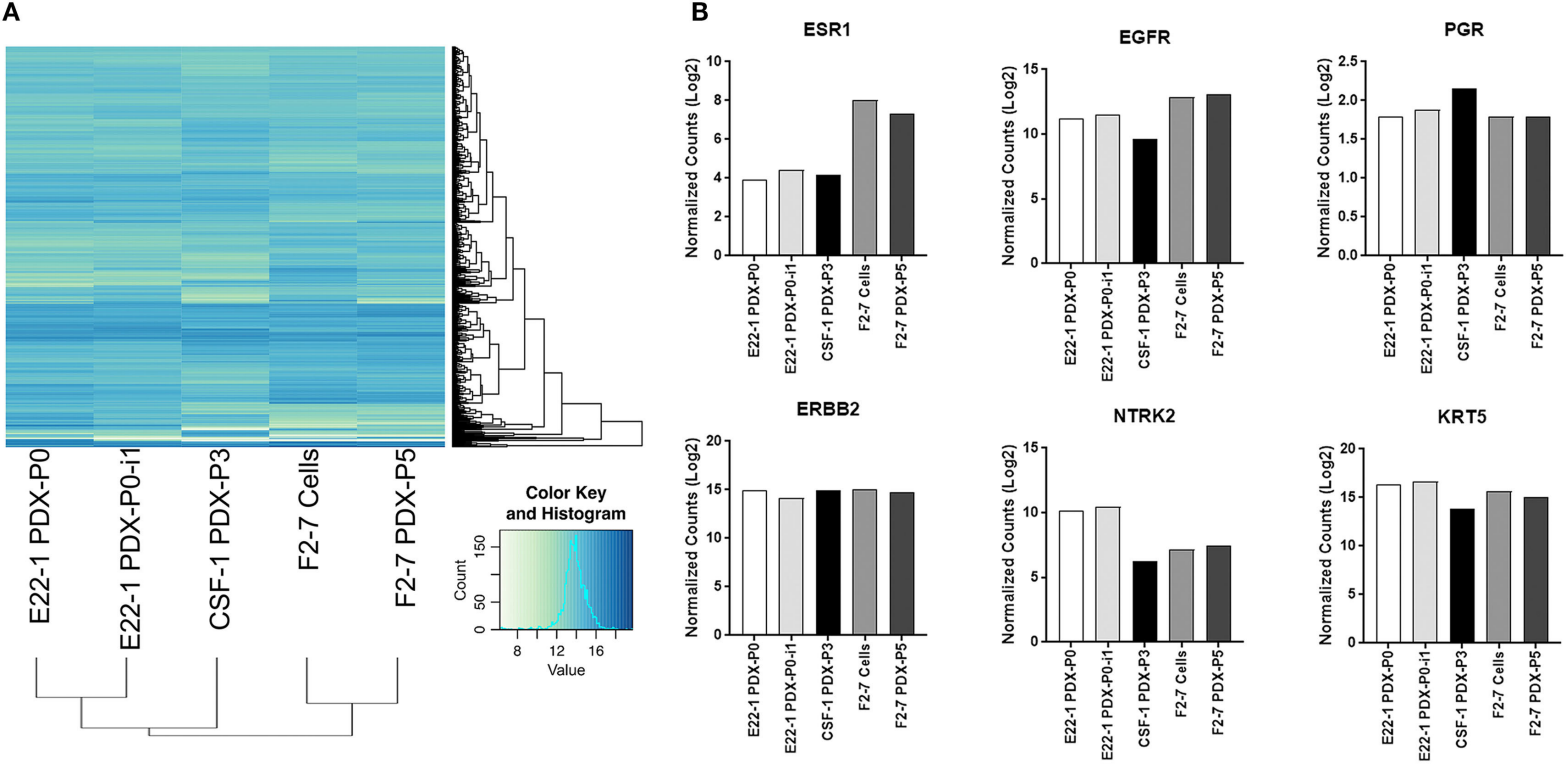
RNA expression profiles of F2-7 cell line and brain-metastases-patient-derived xenografts (BM-PDXs) before and after labeling. RNA sequencing of BM-PDX E22-1 prior to (E22-1-PDX-P0) and after GFP-luciferase labeling (E22-1 PDX-P0-I1), BM-PDX CSF-1 P3, and F2-7 cell-line compared to F2-7 BM-PDX-P5. **(A)** Unsupervised hierarchical clustering of samples using the 500 most variable genes across all samples. **(B)** Normalized expression (log 2) plots of clinically relevant genes (ESR1, EGFR, PGR, ERBB2, NTRK2, KRT5). Differential expression was calculated using cufflinks (cuffdiff), and hierarchical clustering was performed in R.

As tumor-dissociated cells survived short-term *in vitro* culture during labeling, we sought to determine whether dissociated brain-metastatic cells could be cultured as cell lines. We cultured dissociated cells from F2-7, E22, and G5-3 BM-PDXs in plates coated with collagen-I or ultralow attachment plates. In either condition, only cells from F2-7 BM-PDXs survived *in vitro* culture and remained proliferative after multiple passages of repeated freezing and thawing (Figure 4A). This F2-7 cell line-derivative retained expression of EGFR as its BM-PDXs counterpart (Figure 4B), and retained tumor initiating capability *in vitro* (measured as ability to form colonies in the absence of extracellular matrix in mammosphere assays, not shown). To further assess whether BM-PDXs labeling or cell line derivation maintained features of parental BM-PDXs, we performed RNA sequencing followed by hierarchical gene clustering analysis of BM-PDX before and after GFP-luciferase labeling, and F2-7 BM-PDX and its cell line-derivative (Figure 5). Key genes (EGFR, KRT5, NTRK2) were expressed at similar levels in parental PDXs (i.e., E22-1 PDX-P0) compared to its labeled counterpart (E22-1 PDX-P0-I1), and in the F2-7 cell line (F2-7 CL) compared to its BM-PDXs parental (F2-7 P5). Taken together, these data suggests maintenance of clinical markers through passaging and manipulation of BM-PDXs.

### BM-PDXs Retain Their Ability to Colonize the Brain at High Frequencies

**Figure 6:**
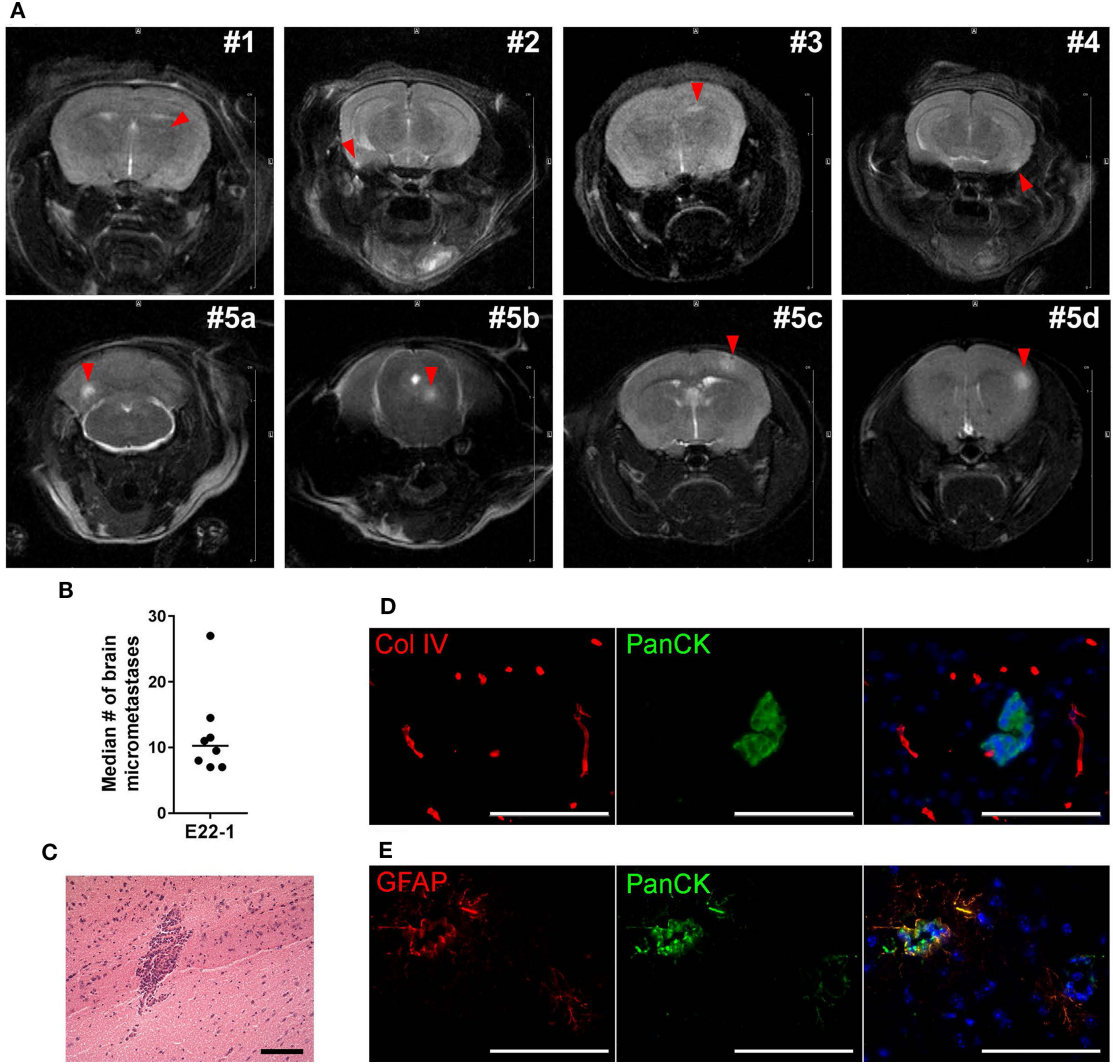
Experimental metastases using brain-metastases-patient-derived xenografts (BM-PDXs). **(A)** Dissociated cells (250,000/100 μl PBS) from BM-PDX E22-1 were injected intracardially in female NSG mice (*n* = 8). Representative T2-weigthed rapid acquisition with relaxation enhancement (RARE) magnetic resonance imaging (MRI) image shows large metastases in 4/10 mice, 8 weeks after injection (top panel). Lower panel shows large and multiple metastases brain metastases in one mouse at 13 weeks postinjection. The summed diameter of all metastases in this mouse was 3.7 mm. **(B)** Median number of micrometastases per mouse, counted in six brain sections, 300 μm apart. **(C)** H&E staining shows micrometastasis in brain section from mice injected with E22-1 BM-PDX. **(D)** Double immunofluorescence staining shows metastatic E22-1 cells (Pan-cytokeratin, PanCK, green) surrounded by reactive astrocytes (GFAP^+^, red) (20×). **(E)** Double immunofluorescence staining shows PanCK^+^E22-1 cells outside of blood vessels (Col-IV) (20×).

Breast cancer PDXs grown in the mammary fat pad rarely metastasize to distant organs, but dissociated cells can colonize lung, bones and brain after ic injection (27). To assess whether BM-PDXs retain their ability to colonize the brain, we induced experimental brain metastasis using dissociated cells from TN E22-1 BM-PDX. For this, 250,000 dissociated cells were injected in the left ventricle of 8–week-old female NSG mice (*n* = 8) supplemented with estradiol, and metastases were allowed to grow until mice showed >15% weight loss or neurological impairment. Nine weeks after ic injection mice were imaged using T1/T2 MRI and mice without symptomatic metastases were left alive for 3 additional weeks. MRI-detectable brain metastases were found in 4/8 (50%) of injected mice, with metastatic lesions ranking from 0.29 to 0.62 mm in size (Figure 6A, top). One additional mouse was injected with E22^−^BM-PDX dissociated cells but MRI was performed 14 weeks after ic injection. This mouse showed multiple large MRI-detectable metastases (Figure 6A, bottom). Histological analysis showed 8/8 mice (100%) harboring micrometastases (defined as >50 μm cancer cell foci counted in six sagittal brain sections 300 μm apart) with a 10.25 median number of micrometastasis per mouse (Figure 6B). To determine whether brain metastases formed by BM-PDXs showed pathophysiological features similar to those encountered in humans, we performed double immunofluorescence staining of brain metastatic cells (pan-cytokeratin^+^, green) and reactive astrocytes (GFAP^+^, red) or blood vessels (Col-IV, red) in brain sections from mice injected with E22-1 BM-PDXs. Brain metastatic clusters were located to the brain parenchyma (Figure 6C), were associated with blood vessels (Figure 6D) and were surrounded by GFAP^+^ reactive astrocytes (Figure 6E); all of these characteristics of breast cancer brain metastases. Taken together, these studies demonstrate that BM-PDXs retain their ability to form large brain metastases and micro metastases at high frequencies, making them suitable models for studies of brain metastatic colonization and preclinical testing of drugs in preventive and therapeutic settings.

## Discussion

**Figure 7:**
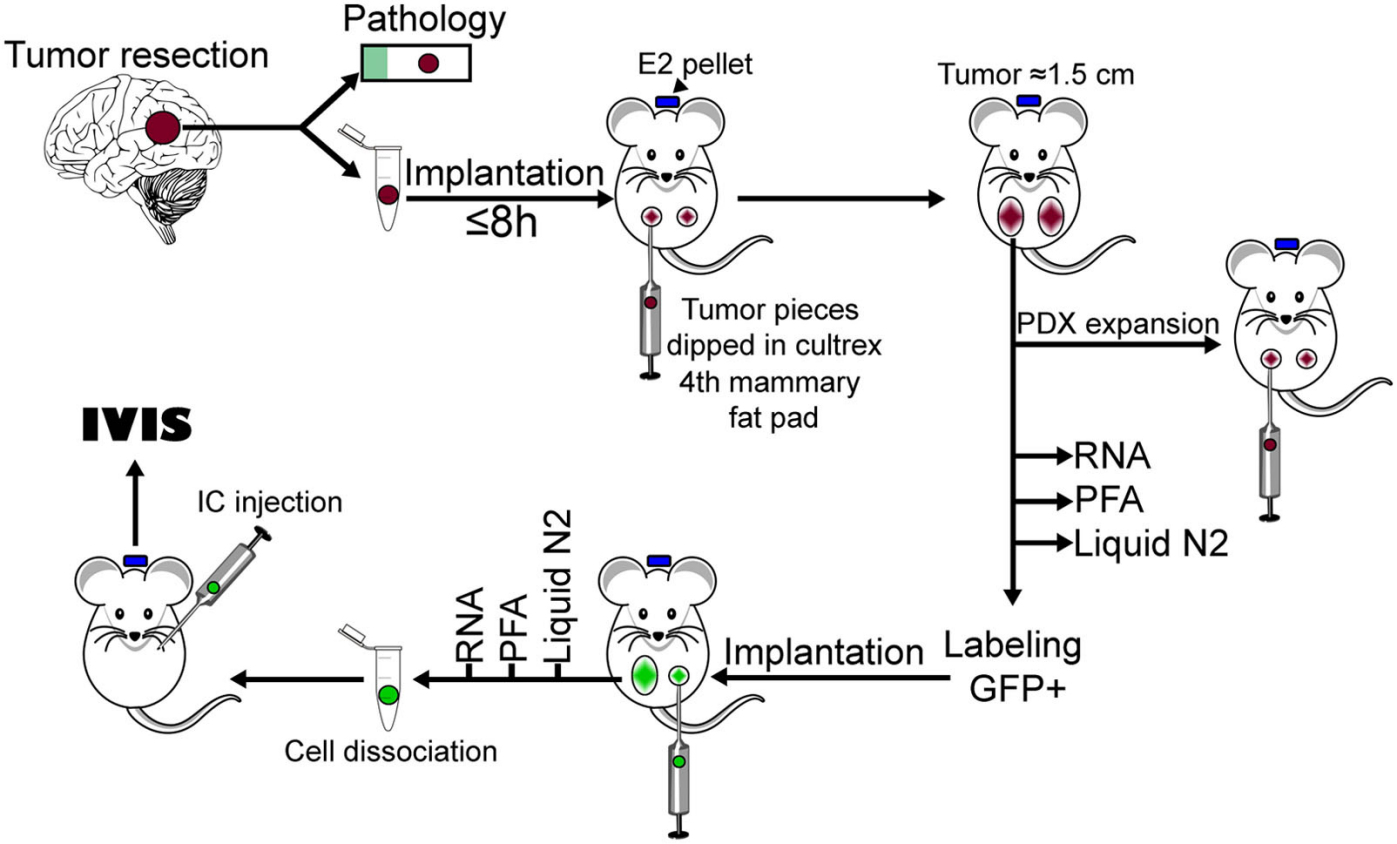
Diagram of procedures used to develop brain-metastases-patient-derived xenografts (BM-PDXs) in this study.

The increased incidence of brain metastasis in breast cancer patients and its dismal prognosis, has prompted the urgency to better understand the pathophysiology of brain metastases and to test novel therapeutic strategies for these patients. PDXs have emerged as required tools to validate *in vitro* studies in cells lines and to decipher the role of tumor heterogeneity in tumor progression and response to treatments (33–35). Therefore, we addressed whether PDX derived from brain-metastatic breast cancer are suitable models to study the pathophysiology of brain metastasis and to provide clinically relevant platforms for therapeutic drug testing. A diagram showing the overall procedure to achieve this from tumor implantation to ic injection of labeled cells is presented in Figure 7. By implanting fresh tumor samples in the mammary fat pad of NSG mice, we developed BM-PDXs from TN, ER^−^HER2^+^, ER^+^HER2^+^, and ER^+^HER2^−^ subtypes. Consistent with the diverse incidence of brain metastasis among breast cancers subtypes (36–38), most specimens collected for implantation originated from TN and HER2^+^ tumors, and 6 of 8 established BM-PDXs were from TN and ER^−^HER2^+^ subtypes. Similarly, BM-PDXs from ER^+^ patients (who show the lowest incidence of BMs) (39) showed the slowest progression when implanted as xenografts (Figure 1B), despite the fact that mice were supplemented with estradiol. Of clinical importance, two specimens had prior history of TNBC but their brain metastasis were reclassified as HER2^+^ by either immunohistochemistry or FISH. This is in agreement with recent reports of ERBB3/HER2 amplifications and mutations in breast cancer brain metastasis that are absent in primary tumors (40). This also highlights how changes in cancer cells occurring within the brain microenvironment modify tumor progression and impact their therapeutic alternatives.

Our BM-PDXs share characteristics of PDX models derived from primary tumors and other cancers. For example, our engraftment rate of 57.3% was similar to rates reported for engraftment of brain metastases from lung cancer (41). We also observed that the *in vivo* tumorigenic potential of patient-derived cancer cells was correlated with worse clinical outcome of patients. This is consistent with the idea that more aggressive/proliferating tumors are more likely to engraft as PDXs (24, 35). Unfortunately, these data also suggest that the potential use of personalized BM-PDX to test drug responses and guide clinical treatment, will not be feasible given the extremely short survival of those brain metastatic patients whose tumors grew as BM-PDXs (Figure 1C).

Similar to other studies, once established as PDXs, tumor cells appear to gain the ability to grow *in vivo* (22, 35), as demonstrated by the shorter time for BM-PDXs to develop into palpable tumors (Figures 1D,E). While no apparent gain or loss of critical cell makers were observed between donor and TN and ER^−^HER2^+^BM-PDXs (Figures 2 and 5), it is possible that the increased growth rate represents differences in the initial number of cancer cells that proliferated to give rise to a PDX, rather than the selection of a subset of rapidly proliferating tumor cells. While we observed a high proportion of cells expressing the basal marker CK5 (a marker associated with a stem-like phenotype in breast cancer) (32, 42, 43), we did not observe enrichment of CK5^+^ after passaging or cell dissociation, which could be interpreted as a selection of a more aggressive tumor clone. However, only genetic tracing of clonal populations within the tumors would allows to answer this question definitively. Our RNA sequencing data showing conserved expression of critical genes after PDX-cell dissociation (Figure 5) suggests that BM-PDXs can be manipulated *in vitro* (i.e., using CRISPR-cas9). This opens the window to use PDXs in mechanistic studies previously limited to cell line models.

Despite being expanded in the mammary fat pad, our BM-PDXs remain capable of colonizing the brain at high frequencies, suggesting that passaging tumors in the mouse does not decrease their brain metastatic potential. While the incidence of MRI-detectable metastases and micrometastases after ic injection were only measured in a cohort of mice injected with the E22-1 BM-PDXs, ongoing experiments in our laboratory suggest that this finding can be extended to the F2-7 cell line and G3-5 BM-PDXs (not shown). Importantly, brain metastases from E22-1 BM-PDX elicit astroglia activation (marked by expression of GFAP^+^ astrocytes) and brain metastatic outgrowth around vessels in the brain parenchyma. Therefore, experimental metastases with BM-PDXs recapitulate interactions with the brain microenvironment recognized as critical for brain metastatic success. Since we can genetically manipulate BM-PDXs dissociated cells or our F2-7 cell line, these novel models are now available to mechanistically assess how diverse breast tumors subtypes adapt to the brain microenvironment. More importantly, since PDXs show a slower progression rate than cell lines, these models are better suited for preclinical testing of drugs in a therapeutic setting, a task difficult to achieve in models where mice become moribund 3–4 weeks after injection.

It has been shown that breast cancer PDXs from primary tumors can colonize the brain if injected ic, suggesting that the intrinsic ability of tumor cells to colonize multiple organs is present in all PDXs regardless of site of origin. While our results indicate that BM-PDXs retain brain tropism, we observed spontaneous metastases of BM-PDXs from the orthotopic site to nearby vessels (Figure 4B) and in a few cases, metastases to bone and lungs after ic injection of BM-PDXs dissociated cells (not shown). This suggests, that similar to brain-homing cell lines and other PDXs, BM-PDXs maintain their ability to disseminate and colonize multiple metastatic sites (44). This also implies that ic injection of BM-PDXs might result in “undesired” metastases to other organs, which will limit our ability to measure brain-metastases-associated survival in these models. Recently, brain-metastatic PDX from lung cancer (41), melanoma (45) and HER2^+^ breast cancer (46) were developed by direct intracranial injection of tumor samples in the brains of mouse or rats. Therefore, direct injection of dissociated cells from BM-PDXs might be an alternative to induce a high frequency of brain metastases while minimizing the confounding effects of peripheral metastases in therapeutic studies. However, direct injection of cancer cells in the brain bypasses critical stages of brain metastastic colonization (hematogenous dissemination, intravasation, neuroinflammatory response, growth around vessels), which are hallmarks of breast cancer brain metastases. Intracarotid artery injection of cancer cells is a suitable alternative to ic injection for the production of brain-only metastasis-bearing mice with similar growth rates and mortality (47). Thus, we propose that intracarotid artery delivery of F2-7 cell line or BM-PDXs dissociated cells will enable the use of these heterogeneous models of brain metastatic breast cancer in mechanistic studies relevant to the pathophysiology of brain metastases, as well as to testing drug efficacy in preventive and therapeutic settings.

In conclusion, we developed and characterized eight novel PDX from breast cancer brain metastases from ER^+^, HER2^+^, and TN subtypes, derived a matching cell line from one TN BM-PDX and demonstrated their brain metastatic potential. While all animal models harbor advantages and limitations, these novel BM-PDXs represent clinically relevant models that can be used to study how the heterogeneity of cancer cells affects brain colonization as well as for validation of therapies.

## Ethics Statement

This study was carried out in accordance with the recommendations of the Department of Health and Human Services (HHS) regulations at 45 CFR 46 (also known as the “Common Rule”) and the Food and Drug Administration (FDA) regulations at 21 CFR 50 and 21 CFR 56; as well as Department of Veterans Affairs policies for human research protection, including the regulations at 38 CFR 16, and the VHA Handbook 1200.05., with written consent from all subject. All subjects gave written informed consent in accordance with the Declaration of Helsinki. The protocol was approved by the Colorado Multiple Institutional Review Board (COMIRB), protocol #13-3007 and the University of Colorado Denver Central Nervous System Biorepository Protocol, Steering Committee.

## Author Contributions

Conception and design: DC. acquisition of data (provided animals, acquired and managed patients, provided facilities, etc.): MC-Z, DO, AG, PK, CH, ND, KL, MG, VB, BJ, SE, AT, and DC. Analysis and interpretation of data (e.g., statistical analysis, biostatistics, imaging, computational analysis): MC-Z, AG, PK, NS, and DC. Administrative, technical, or material support: MC-Z, ND, CH, SE, DO, KL, MG, AT, PK, and DC. Study supervision: DC. All authors contributed to writing, review, and/or revision of the manuscript.

## Conflict of Interest Statement

The authors declare that the research was conducted in the absence of any commercial or financial relationships that could be construed as a potential conflict of interest.

## References

[1] Kaal ECA Niël CGJH Vecht CJ. Therapeutic management of brain metastasis. Lancet Neurol (2005) 4(5):289–98. 10.1016/S1474-4422(05)70072-7 15847842

[2] Lin NU Bellon JR Winer EP CNS metastases in breast cancer. JCO (2004) 22(17):3608–17. 10.1200/JCO.2004.01.175 15337811

[3] Anders C Carey LA Understanding and treating triple-negative breast cancer. Oncology (Williston Park) (2008) 22(11):1233–9; discussion 9-40, 43. 18980022 PMC2868264

[4] Evans AJ James JJ Cornford EJ Chan SY Burrell HC Pinder SE Brain metastases from breast cancer: identification of a high-risk group. Clin Oncol (R Coll Radiol) (2004) 16(5):345–9. 10.1016/j.clon.2004.03.012 15341438

[5] Tham Y-L Sexton K Kramer R Hilsenbeck S Elledge R. Primary breast cancer phenotypes associated with propensity for central nervous system metastases. Cancer (2006) 107(4):696–704. 10.1002/cncr.22041 16826579

[6] Hicks DG Short SM Prescott NL Tarr SM Coleman KA Yoder BJ Breast cancers with brain metastases are more likely to be estrogen receptor negative, express the basal cytokeratin CK5/6, and overexpress HER2 or EGFR. Am J Surg Pathol (2006) 30(9):1097–104. 10.1097/01.pas.0000213306.05811.b9 16931954

[7] Morris PG Murphy CG Mallam D Accordino M Patil S Howard J Limited overall survival in patients with brain metastases from triple negative breast cancer. Breast J (2012) 18(4):345–50. 10.1111/j.1524-4741.2012.01246.x 22607041

[8] Braccini AL Azria D Thezenas S Romieu G Ferrero JM Jacot W. Prognostic factors of brain metastases from breast cancer: impact of targeted therapies. Breast (2013) 22(5):993–8. 10.1016/j.breast.2013.05.011 23831232

[9] Tomasello G Bedard PL de Azambuja E Lossignol D Devriendt D Piccart-Gebhart MJ. Brain metastases in HER2-positive breast cancer: the evolving role of lapatinib. Crit Rev Oncol Hematol (2010) 75(2):110–21. 10.1016/j.critrevonc.2009.11.003 20004109

[10] Ghajar CM Peinado H Mori H Matei IR Evason KJ Brazier H The perivascular niche regulates breast tumour dormancy. Nat Cell Biol (2013) 15(7):807–17. 10.1038/ncb2767 23728425 PMC3826912

[11] Eichler AF Chung E Kodack DP Loeffler JS Fukumura D Jain RK. The biology of brain metastases-translation to new therapies. Nat Rev Clin Oncol (2011) 8(6):344–56. 10.1038/nrclinonc.2011.58 21487419 PMC3259742

[12] Hu G Kang Y Wang XF. From breast to the brain: unraveling the puzzle of metastasis organotropism. J Mol Cell Biol (2009) 1(1):3–5. 10.1093/jmcb/mjp005 19633017

[13] Marino N Woditschka S Reed LT Nakayama J Mayer M Wetzel M Breast cancer metastasis: issues for the personalization of its prevention and treatment. Am J Pathol (2013) 183(4):1084–95. 10.1016/j.ajpath.2013.06.012 23895915 PMC3791679

[14] Palmieri D Smith QR Lockman PR Bronder J Gril B Chambers AF Brain metastases of breast cancer. Breast Dis (2006) 26:139–47. 10.3233/BD-2007-26112 17473372

[15] Palmieri D Bronder JL Herring JM Yoneda T Weil RJ Stark AM Her-2 overexpression increases the metastatic outgrowth of breast cancer cells in the brain. Cancer Res (2007) 67(9):4190–8. 10.1158/0008-5472.CAN-06-3316 17483330

[16] Munoz R Man S Shaked Y Lee CR Wong J Francia G Highly efficacious nontoxic preclinical treatment for advanced metastatic breast cancer using combination oral UFT-cyclophosphamide metronomic chemotherapy. Cancer Res (2006) 66(7):3386–91. 10.1158/0008-5472.CAN-05-4411 16585158

[17] Bos PD Zhang XHF Nadal C Shu W Gomis RR Nguyen DX Genes that mediate breast cancer metastasis to the brain. Nature (2009) 459(7249):1005–9. 10.1038/nature08021 19421193 PMC2698953

[18] Zhang S Huang WC Zhang L Zhang C Lowery FJ Ding Z SRC family kinases as novel therapeutic targets to treat breast cancer brain metastases. Cancer Res (2013) 73(18):5764–74. 10.1158/0008-5472.CAN-12-1803 23913825 PMC3781592

[19] Francia G Cruz-Munoz W Man S Xu P Kerbel RS Mouse models of advanced spontaneous metastasis for experimental therapeutics. Nat Rev Cancer (2011) 11(2):135–41. 10.1038/nrc3001 21258397 PMC4540342

[20] Chen EI Hewel J Krueger JS Tiraby C Weber MR Kralli A Adaptation of energy metabolism in breast cancer brain metastases. Cancer Res (2007) 67(4):1472–86. 10.1158/0008-5472.CAN-06-3137 17308085

[21] Valastyan S Weinberg RA. Tumor metastasis: molecular insights and evolving paradigms. Cell (2011) 147(2):275–92. 10.1016/j.cell.2011.09.024 22000009 PMC3261217

[22] Kabos P Finlay-Schultz J Li C Kline E Finlayson C Wisell J Patient-derived luminal breast cancer xenografts retain hormone receptor heterogeneity and help define unique estrogen-dependent gene signatures. Breast Cancer Res Treat (2012) 135(2):415–32. 10.1007/s10549-012-2164-8 22821401 PMC3818141

[23] DeRose YS Wang G Lin YC Bernard PS Buys SS Ebbert MT Tumor grafts derived from women with breast cancer authentically reflect tumor pathology, growth, metastasis and disease outcomes. Nat Med (2011) 17(11):1514–20. 10.1038/nm.2454 22019887 PMC3553601

[24] Lum DH Matsen C Welm AL Welm BE Overview of human primary tumorgraft models: comparisons with traditional oncology preclinical models and the clinical relevance and utility of primary tumorgrafts in basic and translational oncology research. Curr Protoc Pharmacol (2012) 14:14.22 10.1002/0471141755.ph1422s59 PMC3539738 23258598

[25] Tentler JJ Tan AC Weekes CD Jimeno A Leong S Pitts TM Patient-derived tumour xenografts as models for oncology drug development. Nat Rev Clin Oncol (2012) 9(6):338–50. 10.1038/nrclinonc.2012.61 22508028 PMC3928688

[26] Zhang H Cohen AL Krishnakumar S Wapnir IL Veeriah S Deng G Patient-derived xenografts of triple-negative breast cancer reproduce molecular features of patient tumors and respond to mTOR inhibition. Breast Cancer Res (2014) 16(2):R36. 10.1186/bcr3640 24708766 PMC4053092

[27] Hanna C Kwok L Finlay-Schultz J Sartorius CA Cittelly DM Labeling of breast cancer patient-derived xenografts with traceable reporters for tumor growth and metastasis studies. J Vis Exp (2016) 117:e54944 10.3791/54944 PMC5226329 27929464

[28] Kim D Pertea G Trapnell C Pimentel H Kelley R Salzberg SL. TopHat2: accurate alignment of transcriptomes in the presence of insertions, deletions and gene fusions. Genome Biol (2013) 14(4):R36. 10.1186/gb-2013-14-4-r36 23618408 PMC4053844

[29] Ahdesmaki MJ Gray SR Johnson JH Lai Z Disambiguate: an open-source application for disambiguating two species in next generation sequencing data from grafted samples. F1000Res (2016) 5:2741 10.12688/f1000research.10082.1 27990269 PMC5130069

[30] Liao Y Smyth GK Shi W. The subread aligner: fast, accurate and scalable read mapping by seed-and-vote. Nucleic Acids Res (2013) 41(10):e108. 10.1093/nar/gkt214 23558742 PMC3664803

[31] Love MI Huber W Anders S. Moderated estimation of fold change and dispersion for RNA-seq data with DESeq2. Genome Biol (2014) 15(12):550. 10.1186/s13059-014-0550-8 25516281 PMC4302049

[32] Shao MM Liu J Vong JS Niu Y Germin B Tang P A subset of breast cancer predisposes to brain metastasis. Med Mol Morphol (2011) 44(1):15–20. 10.1007/s00795-010-0495-2 21424932

[33] Rosfjord E Lucas J Li G Gerber HP. Advances in patient-derived tumor xenografts: from target identification to predicting clinical response rates in oncology. Biochem Pharmacol (2014) 91(2):135–43. 10.1016/j.bcp.2014.06.008 24950467

[34] Siolas D Hannon GJ. Patient-derived tumor xenografts: transforming clinical samples into mouse models. Cancer Res (2013) 73(17):5315–9. 10.1158/0008-5472.CAN-13-1069 23733750 PMC3766500

[35] Dobrolecki LE Airhart SD Alferez DG Aparicio S Behbod F Bentires-Alj M Patient-derived xenograft (PDX) models in basic and translational breast cancer research. Cancer Metastasis Rev (2016) 35(4):547–73. 10.1007/s10555-016-9653-x 28025748 PMC5396460

[36] Tseng LM Hsu NC Chen SC Lu YS Lin CH Chang DY Distant metastasis in triple-negative breast cancer. Neoplasma (2013) 60(3):290–4. 10.4149/neo_2013_038 23373998

[37] Sihto H Lundin J Lundin M Lehtimaki T Ristimaki A Holli K Breast cancer biological subtypes and protein expression predict for the preferential distant metastasis sites: a nationwide cohort study. Breast Cancer Res (2011) 13(5):R87. 10.1186/bcr2944 21914172 PMC3262199

[38] Weil RJ Palmieri DC Bronder JL Stark AM Steeg PS. Breast cancer metastasis to the central nervous system. Am J Pathol (2005) 167(4):913–20. 10.1016/S0002-9440(10)61180-7 16192626 PMC1603675

[39] Hung MH Liu CY Shiau CY Hsu CY Tsai YF Wang YL Effect of age and biological subtype on the risk and timing of brain metastasis in breast cancer patients. PLoS One (2014) 9(2):e89389 10.1371/journal.pone.0089389 24586742 PMC3933537

[40] Priedigkeit N Hartmaier RJ Chen Y Vareslija D Basudan A Watters RJ Intrinsic subtype switching and acquired ERBB2/HER2 amplifications and mutations in breast cancer brain metastases. JAMA Oncol (2017) 3(5):666–71. 10.1001/jamaoncol.2016.5630 27926948 PMC5508875

[41] Lee HW Lee JI Lee SJ Cho HJ Song HJ Jeong DE Patient-derived xenografts from non-small cell lung cancer brain metastases are valuable translational platforms for the development of personalized targeted therapy. Clin Cancer Res (2015) 21(5):1172–82. 10.1158/1078-0432.CCR-14-1589 25549722

[42] Sartorius CA Harvell DM Shen T Horwitz KB. Progestins initiate a luminal to myoepithelial switch in estrogen-dependent human breast tumors without altering growth. Cancer Res (2005) 65(21):9779–88. 10.1158/0008-5472.CAN-05-0505 16266999

[43] Cittelly DM Finlay-Schultz J Howe EN Spoelstra NS Axlund SD Hendricks P Progestin suppression of miR-29 potentiates dedifferentiation of breast cancer cells via KLF4. Oncogene (2013) 32(20):2555–64. 10.1038/onc.2012.275 22751119 PMC4236860

[44] Giuliano M Herrera S Christiny P Shaw C Creighton CJ Mitchell T Circulating and disseminated tumor cells from breast cancer patient-derived xenograft-bearing mice as a novel model to study metastasis. Breast Cancer Res (2015) 17:3. 10.1186/s13058-014-0508-5 25572662 PMC4318479

[45] Wang J Daphu I Pedersen PH Miletic H Hovland R Mork S A novel brain metastases model developed in immunodeficient rats closely mimics the growth of metastatic brain tumours in patients. Neuropathol Appl Neurobiol (2011) 37(2):189–205. 10.1111/j.1365-2990.2010.01119.x 20819169

[46] Ni J Ramkissoon SH Xie S Goel S Stover DG Guo H Combination inhibition of PI3K and mTORC1 yields durable remissions in mice bearing orthotopic patient-derived xenografts of HER2-positive breast cancer brain metastases. Nat Med (2016) 22(7):723–6. 10.1038/nm.4120 27270588 PMC4938731

[47] Zhang C Lowery FJ Yu D. Intracarotid cancer cell injection to produce mouse models of brain metastasis. J Vis Exp (2017) 120:e55085. 10.3791/55085 28287553 PMC5409267

